# Using a Consensus Docking Approach to Predict Adverse Drug Reactions in Combination Drug Therapies for Gulf War Illness

**DOI:** 10.3390/ijms19113355

**Published:** 2018-10-26

**Authors:** Rajeev Jaundoo, Jonathan Bohmann, Gloria E. Gutierrez, Nancy Klimas, Gordon Broderick, Travis J. A. Craddock

**Affiliations:** 1 Institute for Neuro-Immune Medicine, Nova Southeastern University, Fort Lauderdale, FL 33314, USA; rj426@nova.edu (R.J.); nklimas@nova.edu (N.K.); Gordon.Broderick@rochesterregional.org (G.B.); 2 Department of Psychology & Neuroscience, Nova Southeastern University, Fort Lauderdale, FL 33314, USA; 3 Department of Clinical Immunology, Nova Southeastern University, Fort Lauderdale, FL 33314, USA; 4 Pharmaceuticals and Bioengineering Department, Southwest Research Institute, San Antonio, TX 78238, USA; jonathan.bohmann@swri.org; 5 Pharmaceuticals and Bioengineering, Chemistry and Chemical Engineering Division, Southwest Research Institute, San Antonio, TX 78238, USA; gloria.gutierrez@swri.org; 6 Miami Veterans Affairs Medical Center, Miami, FL 33125, USA; 7 Centre for Clinical Systems Biology, Rochester General Hospital Research Institute, Rochester, NY 14617, USA; 8 Rochester Institute of Technology, Rochester, NY 14623, USA; 9 Department of Computer Science, Nova Southeastern University, Fort Lauderdale, FL 33314, USA

**Keywords:** Gulf War Illness, consensus docking, off-target interactions, side effects, multi-drug therapy, polypharmacology, treatment course design

## Abstract

Gulf War Illness (GWI) is a chronic multisymptom illness characterized by fatigue, musculoskeletal pain, and gastrointestinal and cognitive dysfunction believed to stem from chemical exposures during the 1990–1991 Persian Gulf War. There are currently no treatments; however, previous studies have predicted a putative multi-intervention treatment composed of inhibiting Th1 immune cytokines followed by inhibition of the glucocorticoid receptor (GCR) to treat GWI. These predictions suggest the use of specific monoclonal antibodies or suramin to target interleukin-2 and tumor necrosis factor α, followed by mifepristone to inhibit the GCR. In addition to this putative treatment strategy, there exist a variety of medications that target GWI symptomatology. As pharmaceuticals are promiscuous molecules, binding to multiple sites beyond their intended targets, leading to off-target interactions, it is key to ensure that none of these medications interfere with the proposed treatment avenue. Here, we used the drug docking programs AutoDock 4.2, AutoDock Vina, and Schrödinger’s Glide to assess the potential off-target immune and hormone interactions of 43 FDA-approved drugs commonly used to treat GWI symptoms in order to determine their putative polypharmacology and minimize adverse drug effects in a combined pharmaceutical treatment. Several of these FDA-approved drugs were predicted to be novel binders of immune and hormonal targets, suggesting caution for their use in the proposed GWI treatment strategy symptoms.

## 1. Introduction

Gulf War Illness (GWI) is a chronic illness with no known cure and affects some 250,000 veterans that have returned from the first Gulf War fought over 25 years ago. GWI is a complex multisymptom disorder that requires long-term treatment and monitoring, which not only places a great financial burden on the patient, but also on the patient’s family, as well on society. Symptoms of GWI include fatigue, musculoskeletal pain, and gastrointestinal and cognitive dysfunctions [1]. The symptomatology of GWI can call for patients to take a variety of medications, including over-the-counter pain relievers or prescription stimulants to relieve fatigue [2]. Furthermore, veterans from the Gulf War may be taking an increasing amount of medications as they age in order to combat age-related comorbid conditions, such as hypertension, hyperlipidemia, or diabetes. Table 1 provides a list of drugs commonly used to treat GWI symptoms.

The combination of medications is increasingly becoming a concern. Pharmaceutical drugs are ‘promiscuous’, with each binding to an average of at least six molecular targets [3]. These ‘promiscuous’ drugs tend to be smaller in size [4], and bind to proteins which share similar gene families [5]. This has the potential to pose serious unintended consequences, as off-target drug interactions can have deleterious side effects, especially when drugs are used in combination. The potential for adverse drug reactions (ADRs) poses a specific problem for the already taxed systems of patients suffering from GWI, who possess a dysregulation of immune signaling tied to stress and sex hormone levels [6,7,8]. These interactions are a normal part of homeostatic regulation, but a ‘promiscuous’ drug could throw this balance off, exacerbating ADRs.

A major hypothesis of GWI pathophysiology suggests the involvement of a neuroinflammatory cascade that was possibly triggered by multiple toxins experienced in the battlefield and then aggravated further by stress [9,10,11], resulting in altered homeostatic regulation. This neuroinflammatory process is consistent with the broad-ranging multitude of GWI symptoms that extend beyond the central nervous system to affect immune and endocrine function. The body’s key stress regulation system, the hypothalamic–pituitary–adrenal (HPA) axis, links the peripheral immune and endocrine systems to the brain and mediates the response to environmental stressors. In support of this hypothesis, HPA axis dysfunction has been reported in GWI [12]. It is important to note, however, that activity of the hypothalamic–pituitary–gonadal (HPG) axis is intertwined with that of the HPA axis, as well as the immune system [13]. For example, the expression of the androgen receptor (AR) has been detected in various immune cell lineages, including neutrophils, mast cells, macrophages, B cells, and T cells [14,15], and androgens are known to enhance CD4+, Th1, and CD8+ cell activity [16].

**Figure 1:**
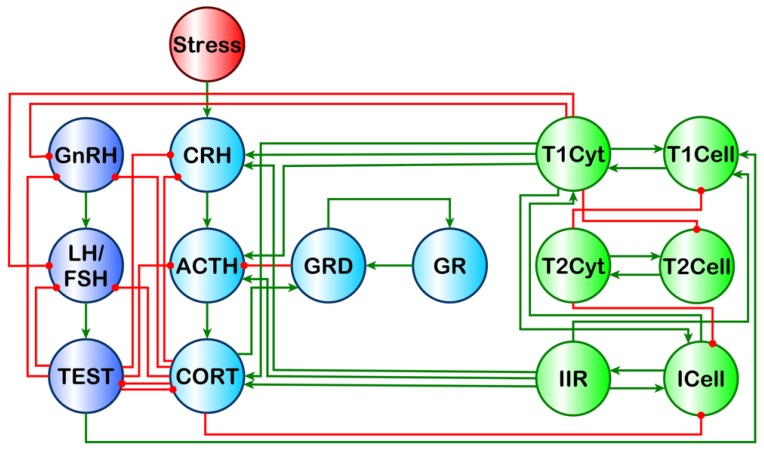
Theoretical male HPA-HPG-immune signaling network. Light blue nodes denote the HPA (hypothalamic–pituitary–adrenal) axis model described by Gupta et al., 2007 [17], comprising corticotropin-releasing hormone (CRH), adrenocorticotropic hormone (ACTH), cortisol (CORT), glucocorticoid receptor (GR), and the dimerized form of the glucocorticoid receptor which follows the binding of CORT (GRD). Dark blue nodes denote the male HPG (hypothalamic–pituitary–gonadal) axis comprised of gonadotropin-releasing hormone (GnRH), luteinizing hormone (LH), follicle-stimulating hormone (FSH), and testosterone (TEST). Green nodes denote a simplified immune system originally, described in [7], comprising innate immune response cytokines (IIR), innate immune cells (ICell), Type 1 T helper cells (T1Cell), Th1 cytokines (T1Cyt), Type 2 T helper cells (T2Cell), and Th2 cytokines (T2Cyt). The red node denotes the external influence of stress on the system. Green edges are stimulatory, and red edges are inhibitory. This image is a reproduction of the original found in [7] and presented under the PLoS ONE Creative Commons Attribution License.

Our previous work aimed to address this issue using a discrete logic model that showed multiple stable homeostatic regulatory behaviors beyond health exist for a simple HPA-HPG-immune network [7] (Figure 1). Of these stable regulatory behaviors, the profile of endocrine–immune balance, measured experimentally in a GWI cohort of male subjects, aligned most closely with a state characterized by elevated cortisol, low testosterone, and a shift towards a Th1 immune response. This alignment, however, does not imply that the homeostatic regulatory drive is the sole cause of GWI, but rather suggests that alignment with this alternate stable regulatory behavior acts in part to sustain the chronicity of this complex illness. These alternate homeostatic regulatory regimes are by definition resistant to change and, therefore, could also promote resistance to therapeutic intervention. Thus, these natural regulatory barriers must be compensated for during the design of any treatment avenue to ensure a robust remission from illness. To this end, our simulations predicted that timed treatments inhibiting Th1 cytokines (interleukin (IL)-2, tumor necrosis factor (TNF)-α, or interferon gamma (IFNγ)) followed by glucocorticoid receptor (GCR) activity would provide the greatest chance to guide the multiple systems from an altered regulatory state back towards healthy behavior with the minimum number of interventions [13]. These same simulations also suggested that the addition of an AR agonist may increase the chance of remission.

While the order and general targets of this proposed treatment course have been stipulated, the specific pharmaceutical combination remains open. This leaves the clinician with a difficult choice when designing clinical trials based on the previously predicted combination therapy, as determining the allowable drug combinations to be used in such a multi-tiered intervention strategy is a nontrivial task, especially when considering the multitude of comorbid conditions associated with aging. Due to the tight regulation between the hormonal and immune pathways [18], the identification of drugs that interact with Th1 cytokines, the AR, and the GCR is required in order to avoid ADRs in such clinical trials. Mifepristone is a known potent GCR antagonist which has previously been used to treat Gulf War veterans with chronic multisymptom illness [19], and the obvious choice for the treatment of Th1 cytokines are monoclonal antibodies which are cytokine-specific [20,21] and may be used to treat chronic inflammatory diseases. That being said, monoclonal antibodies also carry significant risks, such as acute anaphylaxis; serum sickness; and the generation of antibodies [20,22], which is of concern for GWI. Suramin is a small molecule alternative for inhibiting TNF-α [23,24] and IL-2 [25]; however, its side effect of potentially inducing adrenal insufficiency [26] makes it an undesirable choice for this population. From a clinical standpoint, it is key to ensure that there are no small molecules that may interfere with such a risky treatment avenue.

Consistently and reliably predicting the polypharmacologic action of drug agents would be an asset to the healthcare industry for novel drug treatment course development, for the repositioning of United States Food and Drug Administration (FDA)-approved drugs, and for the identification of ADRs. As such, combination treatment design by the clinician should take all precautions to minimize ADRs and off-target interactions, whether for the treatment of a single illness or for the treatment of an illness with comorbid conditions. Here, we characterize FDA-approved drugs commonly used to treat GWI symptoms to find those that have the highest chance of interfering with TNF-α, IL-2, AR, and the GCR in the proposed multidrug GWI treatment course. This was accomplished by performing a consensus docking method using AutoDock 4.2 (AD4) [27], AutoDock Vina 1.1.2 (VINA) [28], and Schrödinger’s Glide 2016-4 (GLIDE) [29] to evaluate potential interactions of 43 FDA-approved small molecule drugs commonly used to treat GWI symptoms (see Table 1) with multiple crystal structures of the GCR and AR in both agonistic and antagonistic forms, as well as the unbound TNF-α and IL-2 cytokines, representing the stress, male sex, and immune components of our previous models, respectively.

## 2. Results

### 2.1. Validation of Docking Accuracy

The ability of the 43 FDA-approved small molecule drugs commonly used to treat GWI symptoms (see Table 1) to interfere with a proposed multidrug GWI treatment course [13] was determined through virtual docking to multiple crystal structures of the GCR, AR, and the TNF-α and IL-2 cytokines. As the GCR and AR both have agonistic and antagonistic forms, we evaluated each of these separately to remove any bias towards a given mode of action in order judge which form of the receptor may be more affected by the 43 GWI symptom-treating drugs. No such difference in forms was available for the TNF-α and IL-2 cytokines. Additionally, we only chose structures that were in complex with a small molecule binder (except 1TNF, see Section 2.3.4 below for clarification); this allowed us to re-dock the known binder using each of the three programs to ensure accuracy. For each target, we only computed results from programs which docked known binders to within a root mean square deviation (RMSD) of 2.0 Å of the crystallographic pose, a value known to reliably identify correctly docked ligands [30]. Table 2 provides a summary of the successes and failures of each program to dock known binders to within the 2.0 Å RMSD cutoff. Note that the crystal structure identifiers refer to targets from the RCSB Protein Data Bank (PDB) [31,32].

**Figure 2:**
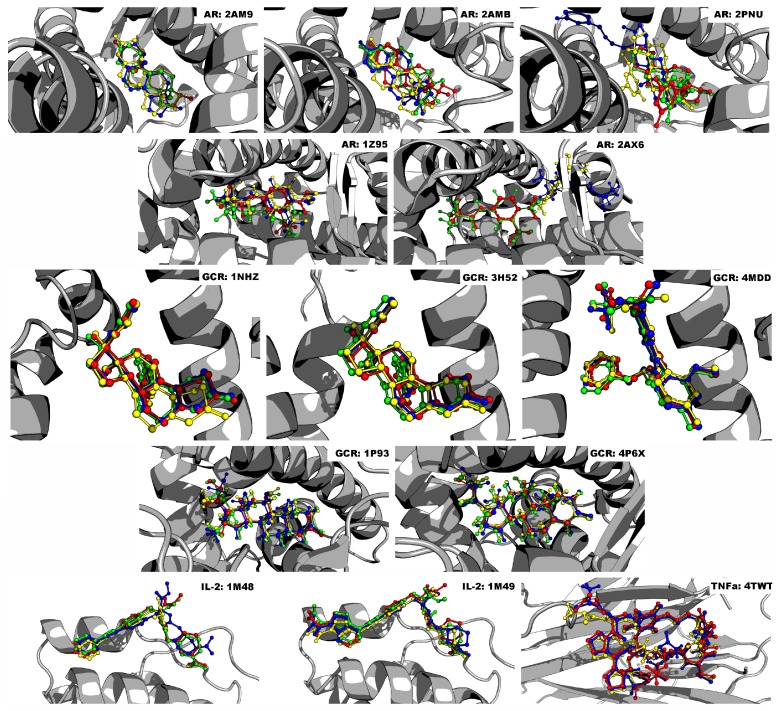
Docked poses of known binders to their targets. Known binder (red) compared to AD4 (yellow), VINA (blue), and GLIDE (green). Note that Residues 636–652 of GCR, and the hydrogen atoms on each ligand, are not shown for clarity.

AD4 and VINA were excluded from AR 2PNU and 2AX6 because their predicted poses for the known binders were above the 2.0 Å RMSD cutoff range. Similar to AD4 and VINA, GLIDE was excluded from AR 2AMB and 1Z95 because it exceeded the RMSD cutoff score. GLIDE failed to predict a pose for TNF-α 4TWT’s known binder altogether. Figure 2 displays the alpha helices and beta sheets of each target’s binding pocket, along with their known binders. The predicted poses from each docking program are shown as well for comparison. Note that all images were created using PyMOL version 1.8.6.2 [33].

### 2.2. Statistical Accuracy

The docking of a ligand with the structure program combinations given in Table 2 yields a distribution of results for each ligand–target interaction. To determine if a given ligand binds to a given target, the results from the various crystal structure–program combinations for the ligand were compared from the distributed results to that of a known binder for the target (i.e., testosterone for AR agonist, hydroxyflutamide for AR antagonist, dexamethasone for GCR agonist, mifepristone for GCR antagonist, and suramin for IL-2 and TNF-α). This comparison was done via a two-sample Student’s t-test with a *p*-value cutoff. To gauge the accuracy of the method, the positive predictive value (PPV), negative predictive value (NPV), sensitivity, and specificity were used to describe the performance of this test of statistical measures through the use of decoys and active compounds obtained from the Database of Useful Decoys: Enhanced [34] for GCR and AR. A total of 50 decoys and 50 active compounds each for AR and GCR in both their agonist and antagonist forms were docked according to Table 2. Those ligands found to have a *p*-value greater than the cutoff (i.e., no statistical difference) were scored as binders, while those below the cutoff were scored as non-binders. Correct comparisons of these results to ligands classified as either an active or inactive compound allowed the tally of true positives or true negatives, respectively, while those that were in disagreement tallied the false negatives and false positives, respectively. These were used to calculate the PPV, NPV, sensitivity, and specificity.

Here, we chose a p-value of 0.02 as our cutoff to obtain the following. For the binding of the 50 active compounds and 50 decoys to the AR agonist forms, the PPV was found to be 92.31% and the NPV was 85.98%, with a sensitivity of 84.85% and a specificity of 92.93%. For the binding of the 50 active compounds and 50 decoys to the AR antagonist forms, the PPV was found to be 75.20% and the NPV was 94.23%, with a sensitivity of 94.00% and a specificity of 75.97%. For binding of the 50 active compounds and 50 decoys to the GCR agonist form, the PPV was found to be 94.57%, and the NPV was 88.68%, with a sensitivity of 87.88% and specificity of 94.95%. For binding of the 50 active compounds and 50 decoys to the GCR antagonist form, the PPV was found to be 100.00% and the NPV was 68.28%, with a sensitivity of 54.00% and specificity of 100.00%.

Grouping all results together yielded a PPV of 86.03%, an NPV of 89.60%, a sensitivity of 88.94%, and a specificity of 86.84% across all AR and GCR targets. The high PPV and NPV indicate that many of the results predicted from this testing procedure are true results. In this regard, there is a high degree of probability that those ligands predicted to be binders are truly binders, whereas there is a lower degree of likelihood that some of the ligands predicted to be non-binders are actually binders. This errs on the side of excluding potential interactions to ensure the reliability of our predictions. Altering the p-value cutoff can change these results to allow for more or less exclusionary criteria.

### 2.3. Docking Results

#### 2.3.1. Glucocorticoid Receptor

##### Antagonist Form

For the GCR antagonist form (Table 3), mifepristone was found to have the lowest mean binding energy of −10.41 ± 0.55 kcal/mol, as expected. This was followed by the other known GCR antagonist 29 M. Comparison of the binding energies predicted for the 43 GWI symptom-treating drugs, 29 M, dexamethasone, cortisol, testosterone, and suramin to those of mifepristone via a *t*-test analysis indicated that all 43 drugs, including the GCR agonist fludrocortisone, and testosterone were significantly lower in binding energy, while the GCR antagonist 29 M was statistically similar to mifepristone, as expected. Dexamethasone and cortisol, known GCR agonists, were also statistically different from mifepristone, as expected from their different modes of action. Unexpectedly, suramin was found to be statistically similar to mifepristone; however, examination of the mean predicted binding energy of suramin indicated a value of 45.87 ± 60.36 kcal/mol. As this value is positive, it does not support suramin binding to the GCR in the antagonistic form; however, the large standard deviation indicates a large discrepancy between the docking program results. Closer inspection of the individual docking results (see Supplementary_Tables.xlsx) shows that while AD4 and VINA both predicted positive binding energies (i.e., no interaction), the GLIDE predicted a binding energy of approximately −7.4 kcal/mol. This suggests that while none of the 43 GWI symptom-treating drugs interfere with the binding of mifepristone, suramin may have the potential for interaction if included in a combination therapy with mifepristone; thus, further investigation of suramin’s interaction with the GCR in the antagonist form is warranted.

##### Agonist Form

For the GCR agonist form (Table 4), dexamethasone was found to have the lowest mean binding energy of −10.41 ± 0.52 kcal/mol, as expected. This was followed by the other known GCR agonists fludrocortisone and cortisol. Comparison of the binding energies predicted for the remaining 42 GWI symptom-treating drugs, testosterone and suramin, to those of dexamethasone via a *t*-test analysis indicated that, beyond fludrocortisone, nefazodone and testosterone were statistically similar in binding energy to dexamethasone, suggesting a potential for agonistic action on the GCR. Suramin, while having a larger mean binding energy than nefazodone, was found to be statistically different from dexamethasone owing to its smaller standard deviation in predicted binding energy values.

#### 2.3.2. Androgen Receptor

##### Agonist Form

For the AR agonist form (Table 5), testosterone was found to have the lowest mean binding energy of −9.61 ± 0.55 kcal/mol, as expected. This was followed by the other known AR agonist, tetrahydrogestrinone, with a binding energy of −9.39 ± 2.27 kcal/mol. The other known agonist of the AR, EM5744, was found to be lower than testosterone and 3 of the 43 drugs used to treat GWI symptoms. However, statistical comparison of results indicates that there is no statistical difference with the testosterone results, as expected. Of the 43 GWI symptom-treating drugs, three were found to have no statistical difference in binding compared to testosterone at a significance level of *p* < 0.02. These three drugs include trazodone, an oral antidepressant used to treat major depressive disorder; carbamazepine, used primarily in the treatment of neuropathic pain; and buspirone, an anxiolytic drug that is primarily used to treat generalized anxiety disorder. Additionally, both suramin and mifepristone were found to be statistically similar to testosterone; however, their mean binding energies were both found to be positive. Unlike suramin interacting with the GCR, GLIDE did not complete runs for these interactions, suggesting no binding interaction, while AD4 and VINA consistently yielded positive binding energies. This indicates that interaction with AR is unlikely, and that the statistical result is due to the large variation in binding energies predicted by the docking programs.

##### Antagonist Form

For the AR antagonist form (Table 6), bicalutamide was found to have the lowest mean binding energy of −8.92 ± 1.52 kcal/mol. The other known antagonist of AR, hydroxyflutamide, was found to have lower binding energy than several of the 43 common GWI treating drugs, as well as suramin and testosterone. Statistical comparison with bicalutamide yielded no significant difference from any of the 43 GWI treating drugs, hydroxyflutamide, suramin, or testosterone, suggesting the unlikely result that all drugs tested, save mifepristone, act on AR in the agonist form. This unlikely prediction is most likely due to the lack of statistical power resulting from having a small number of data points to compare with bicalutamide, as the GLIDE run on 2AX6 failed to complete. As such, statistical comparison was performed using the other known AR antagonist, hydroxyflutamide. Statistical results then yielded no difference from bicalutamide, suramin, or testosterone. Of the 43 GWI symptom-treating drugs, more than half were found to be statistically similar to hydroxyflutamide binding, including naproxen, diclofenac, ibuprofen, and clofibrate, which are known to have AR antagonizing effects [35]. This lack of discrimination is revealed by the relatively low PPV of 75.20% for these comparisons, and is most likely due to hydroxyflutamide having relatively weak predicted binding energies relative to the comparator molecules used to perform statistical tests on the other protein targets in this study.

#### 2.3.3. Interleukin-2

For IL-2 (Table 7), suramin was found to have the lowest mean binding energy of −9.52 ± 1.49 kcal/mol, as expected. This was followed by the known IL-2 binders CMM and FRG with binding energies of −8.44 ± 1.41 kcal/mol and −8.02 ± 0.95 kcal/mol, respectively. Of the 43 GWI symptom-treating drugs, all were found to be statistically different in binding compared to suramin at a significance level of *p* < 0.02. This suggests that none of the 43 GWI symptom-treating drugs interfere with IL-2 activity. Likewise, both mifepristone and testosterone were found to be statistically different from suramin, suggesting no interaction of these drugs with suramin’s effect on IL-2.

#### 2.3.4. Tumor Necrosis Factor-α

Using only the program–structure combinations which successfully docked known crystal structure binders with TNF-α within the RMSD cutoff range of 2.0 Å (see Table 2) resulted in all of the 43 GWI symptom-treating drugs, mifepristone, and testosterone being statistically indistinguishable from suramin at a level of *p* < 0.02. This unlikely prediction is due to the lack of statistical power resulting from having a small number of data points to compare. This is in part due to the TNF-α PDB crystal structure 4TWT being the only one available that met our selection criteria of having a small molecule binder. To alleviate this problem, we relaxed our constraints for TNF-α and included docking results from AD4 and VINA in the TNF-α PDB crystal structure 1TNF.

Using these relaxed conditions, for TNF-α (Table 8), suramin was found to have the lowest mean binding energy of −12.39 ± 3.51 kcal/mol, as expected. This was followed by the other known TNF-α binder M21 with a binding energy of −11.71 ± 3.55 kcal/mol. The binding energies of 34 of the GWI symptom-treating drugs were found to be statistically different from suramin binding at a significance level of *p* < 0.02. The nine drugs found to be statistically similar to suramin binding to TNF-α include buspirone, an anxiolytic drug that is primarily used to treat generalized anxiety disorder; proxetine, an antidepressant of the selective serotonin reuptake inhibitor class; trazodone, an oral antidepressant used to treat major depressive disorder; nefazodone, a serotonin antagonist and reuptake inhibitor related to trazadone; sertraline, another antidepressant of the selective serotonin reuptake inhibitor class; valaciclovir, an antiviral drug; rofecoxib, a nonsteroidal anti-inflammatory drug; gabapentin, a medication used to treat neuropathic pain; and baclofen, a medication used to treat muscle spasticity and pain. Additionally, both mifepristone and testosterone were found to be statistically the same as suramin binding to TNF-α, suggesting the potential for interaction between these drugs and suramin’s effect on TNF-α.

A full collection of all docking binding energy scores for all drugs investigated via each of the docking programs may be found in the Supplementary Tables supplied (Supplementary_Tables.xlsx).

## 3. Discussion

Nearly 250,000 veterans of the 1990–1991 Persian Gulf War suffer daily with a symptom constellation of fatigue, musculoskeletal pain, and gastrointestinal and cognitive dysfunction collectively known as GWI. While there is no widely accepted biomarker for GWI, and afflicted veterans are commonly diagnosed based only on psychological or psychiatric evaluation, the United States Department of Defense (DoD) is moving to alleviate GWI veterans’ suffering (http://cdmrp.army.mil/gwirp/default) through the support of basic and applied research, as well as clinical trials evaluating promising treatments. Recent simulation research based on resetting the homeostatic balance has suggested a multi-tiered intervention strategy aimed at inhibiting Th1 cytokines (such as IL-2 or TNF-α) followed by GCR inhibition [13]. While not a necessity, these same simulations also suggested that the addition of an AR agonist may increase the chance of remission. As clinical trials are currently being developed based on this hypothesized treatment (United States Department of Defense Congressionally Directed Medical Research Program (CDMRP) awards W81XWH-13-2-0085 and GW170044), it is vital for clinicians to be informed about potential interactions with drugs commonly used by veterans with GWI. To this end, the purpose of this work is to identify FDA-approved drugs commonly used to treat symptoms of GWI that may interfere with the proposed Th1 cytokine inhibition, followed by inhibition of the GCR [13]. This was accomplished by using a virtual screening consensus docking approach with AD4, VINA, and GLIDE to evaluate potential interactions of 43 FDA-approved small molecule drugs with multiple structures of the GCR, AR, TNF-α, and IL-2, representing the stress, male sex, and immune components of our previous models, respectively.

Virtual screening is a standard technique used in the drug discovery pipeline [30,36]: drugs are computationally assessed for binding to a specified site on a target based on physicochemical properties in order to determine the ligand’s most likely conformation. The likelihood of the drug–protein interaction is assessed based on the binding energy [30]. That being said, reliable binding energy prediction can be a challenging and difficult task. Program-specific optimization algorithms and scoring functions can often produce highly variable predictions of likely binding poses and ligand affinities to their targets [30]. Consensus docking emends this issue by amalgamating numerous scoring algorithms to reduce error and increase prediction accuracy [30,37,38]. Studies have shown that a consensus docking approach is far more reliable and precise than simply using a single docking method [30,39,40]. Houston and Walkinshaw [30] tested the reliability of such a method, and found that a consensus approach using AD4 and VINA predicted the correct binding pose far more often compared to either of the programs alone. Similarly, other studies have concluded that consensus docking is an improvement over traditional methods for the identification of novel drugs [41,42].

Our main goal was to gauge any potential for interaction of FDA-approved drugs commonly used to treat symptoms of GWI with targets of a proposed multi-intervention treatment strategy for GWI, with the goal to inform the clinician of which drugs to avoid. Clinically, in this case, it is better to err on the side of making false positive predictions, rather than false negatives, as it is better to predict an interaction and exclude the use of a drug with no effect than include one that has interactions. This is opposed to standard rational drug discovery, which aims to rule out all false positives for the purpose of lead development, as including a false positive adds to the overall cost of drug testing and design. Here, we followed the latter principle to ensure the reliability of our predictions at the cost of limiting our overall predictions. However, we provide our results so that erring on the side of false positive predictions can be instituted by allowing more inclusive cutoff criteria (see Supplementary_Tables.xlsx).

Another limiting factor in the standard consensus docking post-processing approach is how to filter results. Filtering is commonly performed by calculating the RMSD between a predicted ligand’s docked pose and the experimental gold standard conformation [30,43,44,45]. If the two poses differ by more than 2.0 Å, then the ligand is removed from further consideration. This method is only useful when the crystallographic pose is available to compare with, and in the case of consensus docking, the RMSDs between each program’s pose are compared. However, if they differ and no crystallographic pose exists, then it is impossible to know which one is correct [30]. Other issues of filtering include too lenient or stringent criteria. Garcia-Sosa and Maran [37] only retained drugs whose binding energy for each docking program was comparable to or lower than the known binder’s energy. This method becomes increasingly exclusive as more docking programs are added. Excessive filtration is especially harmful when important interactions between ligands and targets are overlooked, as in the case of ADRs. For this reason, we opted to include all docking results in the form of a mean binding energy with its standard deviation. The standard deviation provides a measure of consensus across the multiple receptor–program combinations, with a smaller standard deviation indicating better consensus.

The many symptoms of GWI can call for patients to take a variety of medications, such as pain relievers or prescription stimulants to relieve fatigue [2]. Additionally, patients may be taking medications for common comorbidities, such as hypertension and hyperlipidemia. To evaluate the potential for such medications to interact with the proposed multi-intervention Th1-GCR inhibition strategy, we examined the interaction of medications associated with GWI and common comorbidities on the proposed targets of the combined HPA-HPG-immune model. Common pain relief medications include ibuprofen, acetaminophen, diclofenac, and naproxen, among others [2,35]. Atenolol treats high blood pressure or hypertension, clofibrate is a medication for high cholesterol or hyperlipidemia, and carbamazepine is used to prevent and control seizures [35]. In regard to fatigue and/or cognitive dysfunction, amantadine, modafinil, nimodipine are all drugs commonly used for the treatment of these symptoms [2].

In regard to the proposed multi-intervention Th1-GCR inhibition strategy, mifepristone was only found to interact with the GCR in antagonist form, as expected, confirming that it is a reliable, selective GCR antagonist. Testosterone, a suggested supplement to the strategy [13], was found to bind most strongly to the AR in agonist form, as expected, but also showed potential interaction with the AR in antagonist form, as well as the GCR in agonist form. The former may be due to the poorer performance of the AR antagonist predictions, as evidenced by its relatively low PPV. However, since the binding energy of testosterone to the AR in agonist form is much lower than to the antagonist form, this also suggests that if bound to the antagonist form, it may induce a structural change to the agonist form, consistent with expectations. The interaction of testosterone with the GCR in the agonist form, however, cannot be so easily explained. The GCR agonist prediction showed a high PPV of 94.57%, leaving a small chance that the prediction is a false positive. However, it must also be noted that there is evidence that testosterone interferes with dexamethasone binding to the glucocorticoid receptor [46]; however, this is only a weak interaction with the GCR, which is unable to form all the amino acid contacts necessary to yield a stable, transcriptionally active GCR conformation [47]. Our predictions may be reflecting this weak interaction.

In regards to the 43 commonly used medications to treat GWI symptoms, our results suggest that 10 show real potential to interfere with the proposed Th1-GCR multi-inhibition strategy. These include trazadone, buspirone, and carbamazepine with putative interference of AR agonist activity, and buspirone, proxetine, trazodone, nefazodone, sertraline, valaciclovir, rofecoxib, gabapentin, and baclofen with putative interference of TNF-α activity. Use of these drugs should be considered exclusionary criteria for any trial of the Th1-GCR multi-inhibition strategy. Due to the low PPV for the AR antagonist, the potential interactions of the 43 drugs were not considered reliable. However, as naproxen, diclofenac, ibuprofen, and clofibrate were predicted binders to the AR in antagonist form and have been shown to have AR antagonizing effects [35], use of these medications should also be considered as exclusionary criteria in any trial, especially those using a testosterone supplement. While no drugs were found to be statistically comparable to mifepristone binding to the GCR, or with suramin to IL-2, both mifepristone and testosterone were shown to have putative interference with suramin’s effect on TNF-α. As such, it is also not advised to combine suramin with mifepristone or testosterone in the proposed Th1-GCR multi-inhibition strategy. Interference with the AR or TNF-α activity poses a greater concern for such a treatment strategy. The majority of the drugs predicted to have a putative interference with the multi-intervention strategy include medications to treat anxiety and depression. As there has been shown to be a link between inflammation, anxiety, and depression [48], excluding individuals taking these medications from the multi-intervention Th1-GCR inhibition strategy would be prudent. However, it must be noted that the predictions presented in this work have not been experimentally validated. While docking studies such as these can provide useful information about drug–target interaction, they must be experimentally verified to ensure the accuracy of the predictions made. That being said, our goal here is to provide clinicians moving ahead with the proposed Th1-GCR multi-inhibition strategy a gauge for potential drug interactions. It is better to err on the side of caution in this regard.

Our study focused on the prime targets involved in a putative multi-tiered intervention strategy aimed at inhibiting Th1 cytokines (such as IL-2 or TNF-α) followed by GCR inhibition as a first-round assessment of inclusion/exclusion criteria for clinical trials. However, the study is by no means comprehensive. The targets chosen here only represent only a fraction of the HPA-HPG-immune systems. There are many more enzymes, receptors, and targets involved, including multiple immune targets, such as other cytokines and chemokines; other steroid receptors, such as estrogen receptors and mineralcorticoid receptors; as well as other nuclear receptor family members, such as thyroids, PPAR-γ, and liver-X, to name a few. The methods used in this work are not immutable, and may be expanded upon. Docking programs, crystal structures, and drug databases may be substituted or used in addition to our chosen set to account for additional targets, as well as agonist/agonistic forms of the receptors involved. There are no limits to the number of docking programs used; in fact, increasing the number of programs decreases the variance. Our post-processing protocol removes only the most extreme values, such that ligands are ordered based on their average energy. The modularity of the pipeline allows us to investigate a multitude of targets that each implicate numerous pathways without ignoring off-target effects. In this vein, we provide our full dataset in the XLSX file format (see Supplementary_Tables.xlsx) and encourage others to continuously add results from additional docking programs, crystal structures, and additional drugs. This information is not illness-specific and can serve to benefit a multitude of complex diseases by mapping each drug’s interactivity.

## 4. Materials and Methods

### 4.1. Crystal Structure Preparation

Crystal structures of the human AR in agonist (AR: 2AM9, 2AMB, 2PNU) and antagonist (AR: 1Z95, 2AX6) forms, the GCR in agonist (1P93, 4P6X) and antagonist forms (1NHZ, 3H52, 4MDD), IL-2 (1M48, 1M49), and TNF-α (4TWT, 1TNF), were obtained from the PDB [31,32] (http://www.rcsb.org). These crystal structures were chosen based on their amino acid sequence completeness and then their resolution (3 Å or less). Furthermore, we only chose structures that were in complex with a small molecule binder (except 1TNF, see main text for clarification); this allowed us to re-dock the known binder using each of the three programs to ensure accuracy. These crystal structure binders are testosterone for 2AM9, tetrahydrogestrinone for 2AMB, EM5744 ((5S,8R,9S,10S,13R,14S,17S)-13-{2-[(3,5-Difluorobenzyl)oxy]ethyl}-17-hydroxy-10-methylhexadecahydro -3H-cyclopenta[A]phenanthren-3-one for 2PNU, bicalutamide for 1Z95, hydroxyflutamide for 2AX6, dexamethasone for 1P93, cortisol for 4P6X, mifepristone for 1NHZ and 3H52, 29 M (*N*-[2-[benzyl(methyl)amino]methyl-3-(4-fluoro-2-methoxyphenyl)-5-(propan-2-yl)-1*H*-indol-7-yl] methanesulfonamide) for 4MDD, FRG ((*R*)-*N*-[2-[1-(Aminoiminomethyl)-3-piperidinyl] -1-oxoethyl]-4-(phenylethynyl)-*L*-phenylalanine methyl ester) for 1M48, CMM (2-[2-(1-carbamimidoyl- piperidin-3-YL)-acetylamino]-3-{4-[2-(3-oxalyl-1*H*-indol-7-YL)ethyl]-phenyl}-propionic acid methyl ester) for 1M49, and the peptide M21 (ala-cys-pro-pro-cys-leu-trp-gln-val-leu-cys-gly) for 4TWT. Following Garcia-Sosa and Maran’s [37] study, crystal structures for all docking programs were prepared using the Protein Preparation Prepwizard [49,50] tool, which removed waters, added hydrogens, set charges, and adjusted bond orders. Epik [51,52], a pK(a) predictor, was utilized in tandem with Prepwizard and performed tautomerization, as well as predicted ionization states using the Hammett and Taft methodology. The *prepare_target4.py* utility from AutoDockTools 1.5.6 [53] added Gasteiger charges and converted the crystal structures to the format required for AD4 and VINA.

### 4.2. Ligand Preparation

Forty-three FDA-approved ligands commonly used to treat GWI symptoms [2] and drugs known to bind to the targets investigated (i.e., known binders: testosterone, tetrahydrogestrinone, and EM5744 for AR; mifepristone and 29M for GR; suramin, CMM, and FRG for IL-2; and suramin and M21 TNF-α) were obtained from the DrugBank [54,55,56,57] database (accessed on 15 February 2016), and crystal structures from the PDB [31,32] (http://www.rcsb.org). Additionally, 50 active compounds and 50 decoy compounds for the AR, and 50 active compounds and 50 decoy compounds for the GCR were obtained from the Database of Useful Decoys: Enhanced (DUD-E) [34]. No active compounds or decoys were available for IL-2 or TNF-α. The Ligand Preparation [58] tool (LigPrep) was used to add hydrogens, neutralize charged groups, enumerate protonation states using Epik [51,52], and remove waters. These structures were used directly for GLIDE. For AD4 and VINA, the ligands were then converted using the AutoDockTools 1.5.6 [53] utility, *prepare_ligand4.py*, to add Gasteiger charges and produce the format required for AD4 and VINA.

### 4.3. Docking

Virtual screening was performed using the Pegasus supercomputer located at the University of Miami. Drug docking was completed using Python and Bash scripts that implemented Garcia-Sosa and Maran’s [37] protocol for AD4, VINA, and GLIDE. For the docking calculations with Glide, we used the standard precision scoring function for flexible docking. Here, the OPLS3 forcefield was used for post-docking minimization. However, we adjusted the GLIDE protocol so that the Coulomb and van der Waals interaction energy cutoff score (CV cutoff) was set to ‘9999.9’. The inner box size of ‘10.0’ and the outer box size of ‘46.0’ defaults were kept. We performed a single docking run with the more CPU-intensive standard precision scoring function instead of the high-throughput virtual screening function. GLIDE’s scoring functions are based on the amount of CPU time required; high-throughput virtual screening is designed for quick preliminary screenings [59] at the cost of accuracy. On the other hand, standard precision is intended for large databases of drugs [59] and is potentially more accurate, but it also utilizes more computational resources. The settings used for the genetic algorithm in AD4 were: 250 individuals in a population; the settings used for the iterated local search global optimizer based on mutation and local 20,000,000 maximum energy evaluations; 27,000 maximum generations; one individual surviving into next generation; 100 genetic algorithm docking runs; and a ranked cluster analysis was performed on each docking calculation (100 runs of each ligand against each protein). A grid spacing of 0.375 Angstroms was used. The Autodock Binding Energy was used for scoring. These settings are much more intensive than the default AD4 values, but previous studies support their usage in reproducing good scores for known active sites [37]. For VINA, we used an energy range of 3 kcal/mol and eight central processing units (CPUs).

### 4.4. Post-Processing

As there can be a great amount of variation between docking program results, we removed any extreme outliers. We first calculated the median absolute deviation from the median (MADM) of each ligand’s pose from AD4, VINA, and GLIDE from all crystal structures of a given target. The MADM formula is as follows [60]:

(1) $$\mathrm{MADM}=median(|X_{i}-median\left(X\right)|)$$

where *X${}_{i}$* refers to the free binding energy of the pose to the crystal structure, and *X* refers to the median free binding energies from all docking programs from all crystal structures. In contrast to the standard deviation and mean, the MADM is not skewed by outliers, and is able to discern exorbitant values even when the sample size is small [61]. We opted to use the MADM due to this robustness, especially when scoring a wide variety of binding energies. The upper and lower bounds were determined using the formula:

(2) $$\left[X_{lower},X_{upper}\right]=median\left(X\right)\pm \left(3.5\times \mathrm{MADM}\right)$$

Wanting to be as inclusive as possible, we eliminated only extreme binding energies that were greater than a threshold of 3.5 absolute deviations around the median.

### 4.5. Validation of Docking Accuracy

To verify protocol accuracy, all known binders were docked to their respective targets using each docking program. Next, the RMSD between the docked and crystallographic poses were compared, using a cutoff score of 2.0 Å. If any AD4, VINA, or GLIDE failed to dock a known binder within the cutoff score, then the program was omitted for that crystal structure. See Table 2 for details. To accurately compare RMSD, AmberTools16 [62] was first used to normalize atom numbers within the output PDB formatted files for each docking program. For the GCR, the AR, and IL-2, this resulted in at least five program–structure combinations. However, for TNF-α, this only resulted in two program–structure combinations. As such, additional docking runs were performed for TNF-α using GLIDE on 4TWT and AD4 and VINA on 1TNF to accommodate the statistical comparisons discussed below.

### 4.6. Statistical Comparison

As the docking results from the various crystal structure–program combinations yield a statistical distribution, we compared the distributed results for each drug examined to a known binder for a given target (i.e., testosterone for the AR agonist, hydroxyflutamide for the AR antagonist, mifepristone for the GCR antagonist, dexamethasone for the GCR agonist, and suramin for IL-2 and TNF-α). This was done via a two-sample Student’s t-test with a p-value cutoff of 0.02. To gauge the accuracy of this method, the positive predictive value (PPV), negative predictive value (NPV), sensitivity, and specificity were used to describe the performance of our diagnostic test and statistical measures on decoys and active compounds obtained from the Database of Useful Decoys: Enhanced (DUD-E) [34] for AR and GCR.

## Figures and Tables

**Table 1:** Drugs commonly used to treat Gulf War Illness (GWI) symptoms. Adapted from Carruthers et al. [2].

Drug	Symptoms	Mechanism of Action
Acetaminophen	Pain	Unknown
Alprazolam	Anxiety	GABA receptor modulator
Amantadine	Fatigue	Unknown
Amitriptyline	Sleep Disturbance, Pain, Depression	Serotonin norepinephrine reuptake inhibitor
Atenolol	Orthostatic Intolerance	Adrenergic beta receptor antagonist
Baclofen	Pain	GABA receptor agonist
Bupropion	Depression	Norepinephrine-dopamine reuptake inhibitor and nicotinic receptor antagonist
Buspirone	Anxiety	Serotonin 5HT1A receptor agonist
Carbamazepine	Pain	Sodium channel blocker
Celecoxib	Pain	COX inhibiting nonsteroidal anti-inflammatory drug
Citalopram	Depression	Selective serotonin reuptake inhibitor
Clofibrate	Hyperlipidemia	Unknown
Clonazepam	Sleep Disturbance, Anxiety	GABA receptor modulator
Cyclobenzaprine	Sleep Disturbance, Pain	Histamine, serotonin, and muscarinic receptor antagonist
Dextroamphetamine	Fatigue, Cognitive Dysfunction	Trace amine-associated receptor 1 agonist
Diazepam	Anxiety	GABA receptor modulator
Diclofenac	Pain	COX inhibiting nonsteroidal anti-inflammatory drug
Doxepin	Sleep Disturbance, Pain, Depression	Histamine, serotonin, and muscarinic receptor antagonist
Fludrocortisone	Orthostatic Intolerance, HPA Axis Abnormalities	Mineralcorticoid agonist
Fluoxetine	Depression	Selective serotonin reuptake inhibitor
Fluvoxamine	Depression	Selective serotonin reuptake inhibitor and sigma-1 receptor agonist
Gabapentin	Pain	Voltage-dependent calcium channel inhibitor
Ibuprofen	Pain	COX inhibiting nonsteroidal anti-inflammatory drug
Ketorolac	Pain	COX inhibiting nonsteroidal anti-inflammatory drug
l-Tryptophan	Sleep Disturbance	Serotonin and melatonin precursor
Lorazepam	Anxiety	GABA receptor modulator
Meclizine	Vertigo	Histamine receptor antagonist
Methylphenidate	Fatigue, Cognitive Dysfunction	Norepinephrine-dopamine reuptake inhibitor
Midodrine	Orthostatic Intolerance	Precursor for alpha-adrenergic receptor agonist
Modafinil	Fatigue, Cognitive Dysfunction	Dopamine reuptake inhibitor
Naproxen	Pain	COX inhibiting nonsteroidal anti-inflammatory drug
Nefazodone	Depression	Serotonin antagonist and reuptake inhibitor
Nimodipine	Cognitive Dysfunction, High Blood Pressure	Calcium channel blocker
Nortriptyline	Pain, Depression	Histamine, serotonin, and muscarinic receptor antagonist
Oxazepam	Anxiety	GABA receptor modulator
Paroxetine	Orthostatic Intolerance, Depression	Selective serotonin reuptake inhibitor
Pindolol	Orthostatic Introlerance	Adrenergic beta receptor antagonist and serotonin 5-HT1A receptor antagonist
Rofecoxib	Pain	COX inhibiting nonsteroidal anti-inflammatory drug
Sertraline	Depression	Selective serotonin reuptake inhibitor
Trazodone	Sleep Disturbance	Serotonin 5-HT2A receptor agonist and alpha adrenergic receptor antagonist
Valaciclovir	Immune Dysfunction	Precursor for DNA polymerase inhibitor
Venlafaxine	Depression	Serotonin-norepinephrine reuptake inhibitor
Zopiclone	Sleep Disturbance	GABA receptor modulator

**Table 2:** Docking programs that succeeded/failed to produce poses within the root mean square deviation (RMSD) cutoff range of 2.0 Å. * signifies docking programs which succeeded. # indicates supplementary docking runs to support statistical analysis.

Structure	AD4	VINA	GLIDE
**AR agonist** (2AM9)	*	*	*
**AR agonist** (2AMB)	*		
**AR agonist** (2PNU)			*
**AR antagonist** (1Z95)	*	*	
**AR antagonist** (2AX6)			*
**GCR agonist** (1P93)	*	*	*
**GCR agonist** (4P6X)	*	*	*
**GCR antagonist** (1NHZ)	*	*	*
**GCR antagonist** (3H52)	*	*	*
**GCR antagonist** (4MDD)	*	*	*
**IL-2** (1M48)	*	*	*
**IL-2** (1M49)	*	*	*
**TNF-α** (4TWT)	*	*	#
**TNF-α** (1TNF)	#	#	

**Table 3:** Interaction of common drugs used to treat GWI symptoms with the GCR antagonist form. Mean binding energy and standard deviation are in units of kcal/mol. A *t*-test was used to compare to the known binder mifepristone. (+) indicates putative binding, (−) indicates no putative binding, (*) indicates more in-depth analysis required.

Drug	Mean	StDev	*p*-Value	Interaction
Mifepristone	−10.41	0.55	1.00	+
29 M	−8.90	1.91	0.0835	+
Nefazodone	−8.71	0.91	0.00126	−
Trazodone	−8.26	0.26	4.35 × 10${}^{-7}$	−
Meclizine	−8.23	0.21	3.90 × 10${}^{-6}$	−
Buspirone	−8.14	0.67	1.07 × 10${}^{-5}$	−
Testosterone	−7.99	0.69	7.05 × 10${}^{-6}$	−
Lorazepam	−7.96	0.62	2.81 × 10${}^{-6}$	−
Paroxetine	−7.76	0.35	1.25 × 10${}^{-7}$	−
Fludrocortisone	−7.75	0.92	4.09 × 10${}^{-5}$	−
Dexamethasone	−7.75	0.94	1.15 × 10${}^{-5}$	−
Celecoxib	−7.74	0.68	2.06 × 10${}^{-6}$	−
Zopiclone	−7.73	0.41	2.23 × 10${}^{-7}$	−
Alprazolam	−7.66	0.61	7.25 × 10${}^{-7}$	−
Clonazepam	−7.59	0.56	2.66 × 10${}^{-7}$	−
Diazepam	−7.56	1.12	6.72 × 10${}^{-5}$	−
Carbamazepine	−7.52	1.11	5.58 × 10${}^{-5}$	−
Cortisol	−7.49	0.77	1.15 × 10${}^{-6}$	−
Oxazepam	−7.47	0.97	1.46 × 10${}^{-5}$	−
Nortriptyline	−7.40	0.50	5.97 × 10${}^{-8}$	−
Citalopram	−7.35	0.37	7.98 × 10${}^{-9}$	−
Amitriptyline	−7.34	0.73	8.52 × 10${}^{-7}$	−
Cyclobenzaprine	−7.33	0.66	3.60 × 10${}^{-7}$	−
Sertraline	−7.18	0.34	2.67 × 10${}^{-9}$	−
Doxepin	−7.10	0.61	7.71 × 10${}^{-8}$	−
Fluvoxamine	−7.10	0.73	3.57 × 10${}^{-7}$	−
Fluoxetine	−7.05	0.74	3.51 × 10${}^{-7}$	−
Rofecoxib	−7.01	0.30	1.12 × 10${}^{-7}$	−
Ketorolac	−6.98	0.20	8.42 × 10${}^{-9}$	−
Nimodipine	−6.94	0.73	2.04 × 10${}^{-7}$	−
Modafinil	−6.91	0.42	1.27 × 10${}^{-8}$	−
Diclofenac	−6.89	0.43	3.44 × 10${}^{-9}$	−
Pindolol	−6.85	0.82	4.04 × 10${}^{-7}$	−
Valaciclovir	−6.73	0.39	4.74 × 10${}^{-9}$	−
Venlafaxine	−6.32	0.57	3.59 × 10${}^{-9}$	−
Naproxen	−6.19	0.73	1.97 × 10${}^{-8}$	−
Methylphenidate	−6.18	0.13	5.75 × 10${}^{-10}$	−
Baclofen	−6.14	0.54	1.60 × 10${}^{-9}$	−
l-Tryptophan	−6.00	0.18	4.95 × 10${}^{-10}$	−
Atenolol	−5.97	0.78	2.03 × 10${}^{-8}$	−
Midodrine	−5.90	0.36	3.11 × 10${}^{-10}$	−
Ibuprofen	−5.67	0.87	2.53 × 10${}^{-8}$	−
Bupropion	−5.57	0.92	3.32 × 10${}^{-8}$	−
Gabapentin	−5.38	0.45	4.98 × 10${}^{-11}$	−
Clofibrate	−5.25	0.56	1.74 × 10${}^{-10}$	−
Acetaminophen	−5.12	0.77	2.11 × 10${}^{-9}$	−
Amantadine	−4.96	0.26	9.77 × 10${}^{-11}$	−
Dextroamphetamine	−4.60	0.43	1.77 × 10${}^{-9}$	−
Suramin	45.87	60.36	0.04.20	*

**Table 4:** Interaction of common drugs used to treat GWI symptoms with the GCR agonist form. Mean binding energy and standard deviation are in units of kcal/mol. A *t*-test was used to compare to the known binder dexamethasone. (+) indicates putative binding, (−) indicates no putative binding.

Drug	Mean	StDev	*p*-Value	Interaction
Dexamethasone	−10.41	0.52	1.00	+
Fludrocortisone	−10.60	0.70	0.663	+
Cortisol	−10.31	0.77	0.815	+
Testosterone	−9.30	0.67	0.0374	+
Carbamazepine	−8.95	0.36	0.0188	−
Alprazolam	−8.13	0.51	2.98 × 10${}^{-3}$	−
Meclizine	−7.85	0.05	0.0127	−
Suramin	−7.83	0.15	9.71 × 10${}^{-3}$	−
Nefazodone	−7.77	2.46	0.119	+
Ketorolac	−7.71	0.73	8.31 × 10${}^{-4}$	−
Trazodone	−7.70	1.33	3.31 × 10${}^{-3}$	−
Citalopram	−7.68	0.68	8.43 × 10${}^{-4}$	−
Amitriptyline	−7.61	0.74	6.80 × 10${}^{-4}$	−
Buspirone	−7.58	1.99	1.65 × 10${}^{-2}$	−
Valaciclovir	−7.58	0.52	1.36 × 10${}^{-3}$	−
Doxepin	−7.57	0.63	8.27 × 10${}^{-4}$	−
Paroxetine	−7.56	0.35	2.98 × 10${}^{-3}$	−
Sertraline	−7.56	0.33	3.77 × 10${}^{-3}$	−
Nortriptyline	−7.55	0.72	6.26 × 10${}^{-4}$	−
Diclofenac	−7.55	0.62	8.19 × 10${}^{-4}$	−
Fluoxetine	−7.54	0.57	9.70 × 10${}^{-4}$	−
Lorazepam	−7.52	0.18	7.18 × 10${}^{-3}$	−
Rofecoxib	−7.40	0.71	4.94 × 10${}^{-4}$	−
Oxazepam	−7.33	0.77	4.00 × 10${}^{-4}$	−
Cyclobenzaprine	−7.30	0.36	2.14 × 10${}^{-3}$	−
Diazepam	−7.28	0.86	3.68 × 10${}^{-4}$	−
Fluvoxamine	−7.20	0.25	3.97 × 10${}^{-3}$	−
Modafinil	−7.18	0.30	2.85 × 10${}^{-3}$	−
Zopiclone	−7.14	0.48	8.27 × 10${}^{-4}$	−
Clonazepam	−7.03	0.66	3.14 × 10${}^{-4}$	−
Pindolol	−6.94	0.74	2.18 × 10${}^{-4}$	−
Naproxen	−6.88	1.15	3.80 × 10${}^{-4}$	−
Nimodipine	−6.57	0.76	1.21 × 10${}^{-4}$	−
l-Tryptophan	−6.57	0.60	2.39 × 10${}^{-4}$	−
Methylphenidate	−6.54	0.28	2.34 × 10${}^{-3}$	−
Atenolol	−6.48	0.31	1.56 × 10${}^{-3}$	−
Baclofen	−6.38	0.35	1.27 × 10${}^{-3}$	−
Venlafaxine	−6.30	0.29	1.80 × 10${}^{-3}$	−
Midodrine	−6.15	0.29	1.41 × 10${}^{-3}$	−
Ibuprofen	−5.98	0.92	4.86 × 10${}^{-5}$	−
Bupropion	−5.91	1.09	6.74 × 10${}^{-5}$	−
Gabapentin	−5.81	0.35	8.55 × 10${}^{-4}$	−
Amantadine	−5.78	0.06	3.84 × 10${}^{-3}$	−
Clofibrate	−5.73	0.67	6.00 × 10${}^{-5}$	−
Acetaminophen	−5.51	0.55	1.18 × 10${}^{-4}$	−
Dextroamphetamine	−5.24	1.21	4.33 × 10${}^{-5}$	−
Celecoxib	−4.93	1.91	7.53 × 10${}^{-3}$	−
Mifepristone	6.52	7.22	0.0180	−

**Table 5:** Interaction of common drugs used to treat GWI symptoms with AR agonist form. Mean binding energy and standard deviation are in units of kcal/mol. A *t*-test was used to compare to the known binder testosterone. (+) indicates putative binding, (−) indicates no putative binding, (*) indicates more in-depth analysis required.

Drug	Mean	StDev	*p*-Value	Interaction
Testosterone	−9.61	0.55	1.00	+
Tetrahydrogestrinone	−9.39	2.27	0.854	+
Ketorolac	−8.67	0.26	0.0110	−
Paroxetine	−8.37	0.41	0.0110	−
Trazodone	−8.13	2.44	0.278	+
EM5744	−8.07	4.44	0.516	+
Sertraline	−7.93	0.56	5.29 × 10${}^{-3}$	−
Carbamazepine	−7.82	1.14	0.0244	+
Modafinil	−7.74	0.56	1.55 × 10${}^{-3}$	−
Naproxen	−7.73	0.47	8.81 × 10${}^{-4}$	−
Diclofenac	−7.60	0.57	1.11 × 10${}^{-3}$	−
Fluvoxamine	−7.60	1.15	1.92 × 10${}^{-2}$	−
Pindolol	−7.30	0.47	2.45 × 10${}^{-4}$	−
Fludrocortisone	−7.21	0.14	7.96 × 10${}^{-4}$	−
Cyclobenzaprine	−7.17	0.54	2.74 × 10${}^{-4}$	−
Bupropion	−7.16	0.28	9.55 × 10${}^{-4}$	−
Clonazepam	−7.15	0.54	2.01 × 10${}^{-3}$	−
Fluoxetine	−7.07	0.62	3.69 × 10${}^{-4}$	−
Nortriptyline	−6.95	0.21	1.02 × 10${}^{-4}$	−
Citalopram	−6.88	1.14	4.99 × 10${}^{-3}$	−
Doxepin	−6.87	0.85	8.82 × 10${}^{-4}$	−
l-Tryptophan	−6.83	0.55	1.35 × 10${}^{-4}$	−
Rofecoxib	−6.80	1.47	0.0155	−
Baclofen	−6.79	0.20	1.32 × 10${}^{-5}$	−
Ibuprofen	−6.78	0.56	1.26 × 10${}^{-4}$	−
Methylphenidate	−6.74	0.47	6.25 × 10${}^{-5}$	−
Amitriptyline	−6.70	0.65	1.93 × 10${}^{-4}$	−
Atenolol	−6.69	0.49	2.10 × 10${}^{-4}$	−
Venlafaxine	−6.41	0.19	2.22 × 10${}^{-4}$	−
Gabapentin	−6.38	0.37	1.50 × 10${}^{-5}$	−
Midodrine	−6.30	0.95	4.71 × 10${}^{-4}$	−
Clofibrate	−6.29	0.64	7.81 × 10${}^{-5}$	−
Oxazepam	−6.11	2.05	0.0133	−
Buspirone	−6.04	2.31	0.0203	+
Lorazepam	−6.04	1.98	0.0106	−
Valaciclovir	−5.88	0.86	1.40 × 10${}^{-4}$	−
Meclizine	−5.87	0.73	5.60 × 10${}^{-4}$	−
Amantadine	−5.73	1.33	9.83 × 10${}^{-4}$	−
Acetaminophen	−5.47	0.66	2.16 × 10${}^{-5}$	−
Nefazodone	−5.28	1.80	2.50 × 10${}^{-3}$	−
Dextroamphetamine	−5.13	1.80	2.09 × 10${}^{-3}$	−
Diazepam	−5.07	0.17	4.02 × 10${}^{-5}$	−
Alprazolam	−4.78	1.27	2.04 × 10${}^{-4}$	−
Zopiclone	−4.77	3.00	0.0162	−
Nimodipine	−3.42	2.82	5.02 × 10${}^{-3}$	−
Celecoxib	6.95	2.73	2.14 × 10${}^{-5}$	−
Mifepristone	88.45	69.80	0.0335	*
Suramin	1171.30	899.94	0.0419	*

**Table 6:** Interaction of common drugs used to treat GWI symptoms with the AR antagonist form. Mean binding energy and standard deviation are in units of kcal/mol. A *t*-test was used to compare with the known binder hydroxyflutamide. (+) indicates putative binding, (−) indicates no putative binding.

Drug	Mean	StDev	*p*-Value	Interaction
Bicalutamide	−8.92	1.52	0.215	−
Paroxetine	−8.37	0.52	0.0220	+
Suramin	−8.17	0.47	0.0263	+
Ketorolac	−7.85	0.21	0.0102	−
Nefazodone	−7.46	1.92	0.778	+
Diclofenac	−7.41	0.42	0.328	+
Modafinil	−7.30	0.13	0.175	+
Testosterone	−7.15	2.04	0.998	+
Hydroxyflutamide	−7.14	0.07	1	+
Naproxen	−7.03	0.10	0.213	+
Trazodone	−6.99	1.77	0.886	+
Valaciclovir	−6.91	0.43	0.382	+
Atenolol	−6.85	0.77	0.519	+
Sertraline	−6.74	1.24	0.604	+
Fluoxetine	−6.71	0.28	0.0649	+
Cyclobenzaprine	−6.55	1.10	0.403	+
Carbamazepine	−6.54	0.05	1.77 × 10${}^{-3}$	−
Methylphenidate	−6.52	0.02	1.10 × 10${}^{-3}$	−
Buspirone	−6.26	2.40	0.560	+
Fluvoxamine	−6.16	0.05	4.19 × 10${}^{-4}$	−
Baclofen	−6.16	0.51	0.0290	+
Alprazolam	−6.10	0.86	0.104	+
Pindolol	−6.08	1.92	0.393	+
l-Tryptophan	−5.98	1.42	0.230	+
Dextroamphetamine	−5.95	0.35	8.01 × 10${}^{-3}$	−
Rofecoxib	−5.79	1.68	0.222	+
Citalopram	−5.78	1.81	0.249	+
Nortriptyline	−5.68	0.44	4.64 × 10${}^{-3}$	−
Midodrine	−5.63	0.12	4.85 × 10${}^{-5}$	−
Venlafaxine	−5.59	0.27	1.92 × 10${}^{-3}$	−
Gabapentin	−5.58	0.88	0.0371	+
Oxazepam	−5.57	0.90	0.0387	+
Zopiclone	−5.55	2.47	0.307	+
Acetaminophen	−5.31	0.82	0.0183	−
Ibuprofen	−5.28	1.02	0.0405	+
Meclizine	−5.22	1.29	0.0668	+
Amantadine	−5.18	0.03	3.84 × 10${}^{-5}$	−
Fludrocortisone	−5.18	3.22	0.332	+
Amitriptyline	−5.15	0.27	2.49 × 10${}^{-4}$	−
Clofibrate	−5.14	1.08	0.0391	+
Diazepam	−5.12	0.74	0.0140	−
Doxepin	−5.09	0.35	5.37 × 10${}^{-4}$	−
Bupropion	−5.01	0.01	2.69 × 10${}^{-5}$	−
Clonazepam	−4.82	0.87	0.000150	−
Nimodipine	−4.58	0.68	5.75 × 10${}^{-3}$	−
Lorazepam	−4.36	0.27	6.60 × 10${}^{-5}$	−
Celecoxib	−3.22	1.72	0.0227	+
Mifepristone	9.77	2.78	1.40 × 10${}^{-3}$	−

**Table 7:** Interaction between common drugs used to treat GWI symptoms and IL-2. Mean binding energy and standard deviation are in units of kcal/mol. A *t*-test used to compare with the known binder suramin. (+) indicates putative binding, (−) indicates no putative binding.

Drug	Mean	StDev	*p*-Value	Interaction
Suramin	−9.52	1.49	1.00	+
CMM	−8.44	1.41	0.333	+
FRG	−8.02	0.95	0.162	+
Nefazodone	−7.15	0.12	0.0141	−
Trazodone	−6.99	0.15	0.0103	−
Meclizine	−6.93	0.10	9.12 × 10${}^{-3}$	−
Mifepristone	−6.50	0.23	4.23 × 10${}^{-3}$	−
Buspirone	−6.24	1.64	4.66 × 10${}^{-3}$	−
Testosterone	−6.01	0.33	1.84 × 10${}^{-3}$	−
Fluoxetine	−5.84	0.14	1.29 × 10${}^{-3}$	−
Zopiclone	−5.78	0.26	1.21 × 10${}^{-3}$	−
Baclofen	−5.78	0.83	3.11 × 10${}^{-4}$	−
Fludrocortisone	−5.72	0.15	1.06 × 10${}^{-3}$	−
Citalopram	−5.69	0.22	1.02 × 10${}^{-3}$	−
l-Tryptophan	−5.61	0.40	9.87 × 10${}^{-5}$	−
Amitriptyline	−5.59	0.15	8.49 × 10${}^{-4}$	−
Ketorolac	−5.57	0.59	1.23 × 10${}^{-4}$	−
Pindolol	−5.56	0.62	1.26 × 10${}^{-4}$	−
Doxepin	−5.55	0.20	8.09 × 10${}^{-4}$	−
Nortriptyline	−5.53	0.78	4.41 × 10${}^{-4}$	−
Diclofenac	−5.47	0.76	1.44 × 10${}^{-4}$	−
Carbamazepine	−5.46	0.68	3.33 × 10${}^{-4}$	−
Rofecoxib	−5.38	0.69	8.87 × 10${}^{-4}$	−
Paroxetine	−5.24	1.60	7.19 × 10${}^{-4}$	−
Methylphenidate	−5.23	0.51	1.76 × 10${}^{-4}$	−
Alprazolam	−5.22	0.94	1.33 × 10${}^{-4}$	−
Modafinil	−5.22	0.13	4.75 × 10${}^{-4}$	−
Lorazepam	−5.15	0.85	9.53 × 10${}^{-5}$	−
Sertraline	−5.10	0.87	9.01 × 10${}^{-5}$	−
Fluvoxamine	−5.07	1.55	4.76 × 10${}^{-4}$	−
Oxazepam	−5.04	0.54	4.03 × 10${}^{-5}$	−
Clonazepam	−5.01	0.44	1.11 × 10${}^{-4}$	−
Bupropion	−4.94	0.14	3.14 × 10${}^{-4}$	−
Cyclobenzaprine	−4.92	1.10	1.17 × 10${}^{-4}$	−
Celecoxib	−4.90	1.09	1.09 × 10${}^{-4}$	−
Atenolol	−4.76	1.32	1.57 × 10${}^{-4}$	−
Dextroamphetamine	−4.74	0.28	1.55 × 10${}^{-5}$	−
Naproxen	−4.69	0.16	2.22 × 10${}^{-4}$	−
Acetaminophen	−4.66	0.64	2.40 × 10${}^{-5}$	−
Ibuprofen	−4.65	0.52	1.88 × 10${}^{-5}$	−
Venlafaxine	−4.64	0.29	1.32 × 10${}^{-5}$	−
Midodrine	−4.62	0.14	1.98 × 10${}^{-4}$	−
Diazepam	−4.57	0.10	1.84 × 10${}^{-4}$	−
Amantadine	−4.27	0.67	1.82 × 10${}^{-4}$	−
Clofibrate	−4.26	0.37	7.59 × 10${}^{-6}$	−
Valaciclovir	−4.25	0.30	1.29 × 10${}^{-4}$	−
Nimodipine	−4.23	0.59	1.05 × 10${}^{-5}$	−
Gabapentin	−3.70	0.15	5.98 × 10${}^{-5}$	−

**Table 8:** Interaction between common drugs used to treat GWI symptoms and TNF-α. Mean binding energy and standard deviation are in units of kcal/mol. A *t*-test was used to compare with the known binder suramin. (+) indicates putative binding, (−) indicates no putative binding.

Drug	Mean	StDev	*p*-Value	Interaction
Suramin	−12.39	3.51	1.00	+
M21	−8.00	0.85	0.195	+
Testosterone	−7.39	0.59	0.108	+
Mifepristone	−7.19	0.30	0.0668	+
Buspirone	−7.18	1.10	0.0306	+
Paroxetine	−6.71	1.28	0.0246	+
Trazodone	−6.60	0.98	0.0244	+
Nefazodone	−6.56	1.49	0.0245	+
Sertraline	−6.10	0.28	0.0291	+
Lorazepam	−6.08	0.04	0.0198	−
Atenolol	−5.96	0.93	0.0150	−
Valaciclovir	−5.93	0.74	0.0241	+
Fludrocortisone	−5.89	0.13	0.0134	−
Pindolol	−5.88	0.95	0.0157	−
Meclizine	−5.87	0.18	0.0136	−
Nortriptyline	−5.83	0.11	0.0193	−
Ketorolac	−5.81	0.27	0.0132	−
Fluvoxamine	−5.76	1.35	0.0102	−
Carbamazepine	−5.75	0.50	0.0179	−
Citalopram	−5.74	0.20	0.0159	−
Zopiclone	−5.64	0.34	0.0137	−
Celecoxib	−5.62	0.54	0.0124	−
Modafinil	−5.62	0.16	0.0108	−
Oxazepam	−5.62	0.54	0.0150	−
Clonazepam	−5.61	0.70	0.0123	−
Cyclobenzaprine	−5.56	0.06	0.0187	−
Amitriptyline	−5.55	0.36	9.49 × 10${}^{-3}$	−
Doxepin	−5.54	0.09	9.91 × 10${}^{-3}$	−
Methylphenidate	−5.40	0.28	8.89 × 10${}^{-3}$	−
Diazepam	−5.37	0.75	0.0131	−
l-Tryptophan	−5.37	0.37	0.0112	−
Rofecoxib	−5.34	0.51	0.0264	+
Midodrine	−5.33	0.88	9.19 × 10${}^{-3}$	−
Diclofenac	−5.27	0.05	0.0117	−
Alprazolam	−5.25	0.36	0.0119	−
Fluoxetine	−5.15	0.35	7.15 × 10${}^{-3}$	−
Gabapentin	−5.01	0.72	0.0217	+
Baclofen	−5.01	0.86	0.0305	+
Naproxen	−4.70	0.71	7.37 × 10${}^{-3}$	−
Amantadine	−4.66	0.08	0.0142	−
Venlafaxine	−4.61	0.01	5.52 × 10${}^{-3}$	−
Acetaminophen	−4.41	0.01	0.0130	−
Clofibrate	−4.34	0.06	0.0104	−
Nimodipine	−4.31	0.27	7.96 × 10${}^{-3}$	−
Dextroamphetamine	−4.08	0.17	9.59 × 10${}^{-3}$	−
Bupropion	−3.97	0.47	4.85 × 10${}^{-3}$	−
Ibuprofen	−3.80	1.14	4.13 × 10${}^{-3}$	−

## Supplementary Materials

Supplementary materials are available online at http://www.mdpi.com/1422-0067/19/11/3355/s1.

## Abbreviations

29 M	*N*-[2-{[benzyl(methyl)amino]methyl}-3-(4-fluoro-2-methoxyphenyl)-5-(propan-2-yl)-1*H*-indol-7-yl]
	methanesulfonamide
AD4	AutoDock 4.2
ADR	adverse drug reaction
AR	androgen receptor
CMM	2-[2-(1-carbamimidoyl-piperidin-3-YL)-acetylamino]-3-{4-[2-(3-oxalyl-1*H*-indol-7-YL)ethyl]-phenyl}-
	propionic acid methyl ester
EM5744	(5S,8R,9S,10S,13R,14S,17S)-13-{2-[(3,5-Difluorobenzyl)oxy]ethyl}-17-hydroxy-10-
	methylhexadecahydro-3*H*-cyclopenta[A]phenanthren-3-one
FDA	United States Food and Drug Administration
FRG	(*R*)-*N*-[2-[1-(Aminoiminomethyl)-3-piperidinyl]-1-oxoethyl]-4-(phenylethynyl)-l-phenylalanine methyl ester
GCR	glucocorticoid receptor
GLIDE	Schrödinger’s Glide 2016-4
GWI	Gulf War Illness
HPA	hypothalamic–pituitary–adrenal
HPG	hypothalamic–pituitary–gonadal
IL	interleukin
M21	ala-cys-pro-pro-cys-leu-trp-gln-val-leu-cys-gly
MADM	median absolute deviation from the median
NPV	negative predictive value
PDB	Protein Data Bank
PPV	positive predictive value
RMSD	root mean square deviation
TNF	tumor necrosis factor
VINA	AutoDock Vina 1.1.2
